# Whole exome sequencing reveals novel somatic alterations in neuroblastoma patients with chemotherapy

**DOI:** 10.1186/s12935-018-0521-3

**Published:** 2018-02-17

**Authors:** Chao Duan, Han Wang, Ying Chen, Ping Chu, Tianyu Xing, Chao Gao, Zhixia Yue, Jie Zheng, Mei Jin, Weiyue Gu, Xiaoli Ma

**Affiliations:** 1 Beijing Key Laboratory of Pediatric Hematology Oncology, National Discipline of Pediatrics, Ministry of Education, MOE Key Laboratory of Major Diseases in Children, Hematology Oncology Center, Beijing Children’s Hospital, Capital Medical University, National Center for Children’s Health, No. 56 Nanlishi Road, Beijing, 100045 China; 2 Joy Orient Translational Medicine Research Center for the Sequences Analysis and Blast, Beijing, China

**Keywords:** BPTF, Chemotherapy, Neuroblastoma, Novel somatic alterations, Whole exome sequencing

## Abstract

**Background:**

We ought to explore the acquired somatic alterations, shedding light on genetic basis of somatic alterations in NB patients with chemotherapy.

**Methods:**

Marrow blood samples from NB patients were collected before treatment, after the 2nd and 4th chemotherapy for baseline research and continuous monitoring by whole exome sequencing. Plasma cell free DNA (cfDNA) was prepared for baseline research. Finger nail cells were extracted as self control. The clinical data was analyzed.

**Results:**

From December 2014 to February 2016, 27 cases of children with stage IV NB were diagnosed. The follow up time ranged from 5 to 25 months, with a median follow up time of 17 months, 20 patients were stable, one patient died of pulmonary embolism during surgery, six patients died of disease progression. Marrow blood whole exome sequencing demonstrated that several novel somatic mutations were identified in all three trios comply or against the trendy of tumor size variation. Of note, six recurrent mutations in bromodomain PHD finger transcription factor (BPTF) were identified in nine NB patients under the continuous monitoring. The mutation rates variation was positively correlated to tumor size (CC = 0.428, P = 0.021), and patients with BPTF mutation may have a worse prognosis compared with wild type. Meanwhile, CGREF1, CUX2, GP1BA, SLC45A1 and TRA2A were mutated with the trendy oppose as therapeutic effects. The baseline research in three NB patients demonstrated that mutation rate of BPTF, TMCO3, GPRIN2 and C20orf96 in plasma cfDNA were in positive correlation with bone marrow genomic DNA (P = 0.001).

**Conclusions:**

Our study showed that BPTF along with other mutations may function as a biomarker for evaluating to effects of chemotherapy to this refractory tumor, and patients with BPTF mutation might have a worse prognosis.

**Electronic supplementary material:**

The online version of this article (10.1186/s12935-018-0521-3) contains supplementary material, which is available to authorized users.

## Background

Neuroblastoma (NB) is a pediatric malignancy affects young children and responsible for approximately 10–12% of childhood cancer mortality [1]. It originals from the neural crest precursor cells of the developing sympathetic nervous system and is remarkably heterogenous in its malignant potential with diverse clinical presentations, courses, and overall prognosis. Although notably improvements have been achieved in the children with lower-risk NB in the past decades, children suffered with higher stage disease remains to be incurable with survival rates of less than 50%. Although 1p deletion, N-myc amplification, or gain of 17q may identify subtypes of NB and impact survival, yet there is no common NB-specific genomic alteration were uncovered [2].

Here we used whole exome sequencing (WES) to continuous screening the genetic somatic alteration in the NB individuals under the chemotherapy. We ought to explore the acquired somatic alteration, shedding light on genetic basis of somatic alteration in NB patient with chemotherapy.

## Method

### Patients and samples

From December 2014 to February 2016, 27 cases of children with stage IV NB were diagnosed, according to the international neuroblastoma staging system (INSS). Considering the age and N-myc gene status, all of the 27 patients were in high-risk group. In addition, bone marrow aspirates, trephine biopsy and N-myc copies were obtained. And treated by multimodal therapy combined with surgery, chemotherapy, and autologous stem cell transplantation based on the Beijing Children’s Hospital (BCH)-NB-2007 protocol [3–5]. BCH-NB-2007 protocol for high-risk neuroblastoma (based on Hong Kong N6 protocol), consisted of high dose cyclophosphamide, adriamycin and vincristine (CAV) and high dose cisplatinum and VP16 (CVP). The chemotherapy included seven cycles, with cycle 1, 2, 4 and 6 with CAV, and cycle 3 and 7 with CVP. Chemotherapy was performed every 21 days. Therapeutic effect was evaluated through images and tumor markers. Patients were followed up to March 31, 2017.

The trios “diagnosis-2nd chemotherapy-4th chemotherapy” was enrolled to uncover the somatic genetic alterations during the chemotherapy. Marrow blood samples from NB patients were collected before treatment, after the 2nd and 4th chemotherapy for baseline research and continuous monitoring by WES. Plasma cell free DNA (cfDNA) was prepared by Serum/Plasma Circulating DNA Kit (DP339, TIANGEN) for baseline research. Finger nail cells were extracted as self control.

The study was approved by the local ethics committee and written informed consent was obtained from the parents or guardians of each patient in accordance with the Helsinki protocol.

### Next-generation sequencing (NGS) and DNA sequence analysis

To determine the somatic genetic alteration of NB patients under chemotherapy, we selected the time point of diagnosis, 2nd chemotherapy and 4th chemotherapy for further investigation. To uncover the tumor-specific genes in NB individuals, we tracked the mutation rate variation in the process of chemotherapy and evaluated the relationship between these genes and treatment responses. Genomic DNA from NB patients were sheared by sonication. Nine tumor recurrence resistance related genes including BPTF, TMCO3, TH, PHOX2B, DCX, CGREF1, CUX2, SLC45A1, and TRA2A were sequenced.

The sheared genomic DNA was then hybridized with a NimbleGen probe capture array. The libraries were first tested for enrichment by qPCR and for size distribution and concentration using the Agilent Bioanalyzer 2100. The samples were then sequenced on an Illumina Hiseq 2500. Two parallel reactions were done for each sample.

### Data filtering, mapping and variant detection

Raw image files were processed by the BclToFastq (Illumina) for base calling and generating the raw data. The low-quality variations were filtered out using the quality score ≥ 20 (Q20). The sequencing reads were aligned to the NCBI human reference genome (hg19) using BWA. Samtools and Pindel were used to analyzed SNP and indel of the sequence (Additional file 1).

## Results

### Clinical characteristics of 27 patients with HR-NB

27 cases of children with stage IV NB, consisted of 15 females and 12 males, with a median age of 39 months (range 15–101 months), courses varied from 20 days to 5 months. The primary sites were renicapsule and retroperitoneum for 24 and posterior mediastinum for three patients. Distant metastasis involved multi-bones, skull, pancreas, spleen, kidney, pleura, lymph nodes and soft tissue. Bone marrow metastasis for 21 and N-myc amplification for 13 patients. All patients under went multimodal therapy including induction chemotherapy, surgery, megatherapy, radiotherapy and 13-*cis*-retinoic acid maintenance. The response evaluation of the primary tumor after 2nd and 4th cycles of chemotherapy showed different degrees of tumor reduction (Table 1).

**Table 1:** Clinical characteristics and outcome of 27 patients with high-risk neuroblastoma

No.	Age Month	Gender	Primary site	BM	N-MYC amplification	Tumor reduction		FU	Outcome
						RE1	RE2 (%)		
1	39	F	R	Y	N	80%	94	14	CR
2	28	F	R	Y	Y	53%	63	17	Dead
3	30	M	R	Y	Y	94%	99	12	Dead
4	24	F	R	Y	Y	70%	91	19	CR
5	84	M	R	Y	Y	0%	0	17	PR
6	38	F	R	Y	Y	51%	60	20	PR
7	61	F	R	Y	N	41%	64	16	PR
8	36	M	P	Y	Y	79%	79	18	PR
9	39	F	R	Y	Y	72%	77	18	PR
10	19	F	R	N	N	88%	93	21	PR
11	78	M	R	Y	N	70%	89	25	PR
12	30	M	R	N	Y	47%	82	14	Dead
13	40	F	R	N	N	49%	57	13	PR
14	19	F	R	N	N	63%	50	6	Dead
15	59	F	R	Y	N	86%	99	22	CR
16	49	M	P	N	Y	60%	97	13	Dead
17	39	M	R	Y	N	75%	86	16	PR
18	48	M	R	Y	Y	29%	35	18	PR
19	23	F	R	Y	Y	25%	50	5	Dead
20	54	F	R	Y	N	50%	60	13	PR
21	69	F	R	Y	N	60%	80	14	VGPR
22	41	F	R	N	N	0	22	15	PR
23	101	M	P	Y	N	0	15	20	Stable
24	61	M	R	Y	Y	71%	92	20	CR
25	36	F	R	Y	N	50%	81	19	VGPR
26	15	M	R	Y	N	67%	96	21	CR
27	31	M	R	Y	Y	71%	95	14	Dead

*R* retroperitoneum and renicapsule, *P* posterior mediastinum, *FU* follow up

The follow up time ranged from 5 to 25 months, with a median follow up time of 17 months, 20 patients were stable, one patient died of pulmonary embolism during surgery, six patients had progressive diseases, all of six patients died.

### WES in the NB patients under chemotherapy process

WES was performed in three trios of “diagnosis-2nd chemotherapy-4th chemotherapy” patient sample. Two samples on 2nd and 4th chemotherapy time point was excluded due to failing to extract genomic DNA. The average of GC content is 43.67% and the Q20 is more than 95%. Quality control files demonstrate that the data is reliable and adequate for further analysis.

**Fig. 1:**
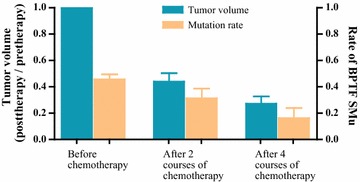
Correlation of BPTF gene mutation rate tumor volume during chemotherapy: correlation analysis showed that the BPTF gene mutation rate was in positive correlation with tumor volume during chemotherapy, (CC = 0.428, P = 0.021)

**Fig. 2:**
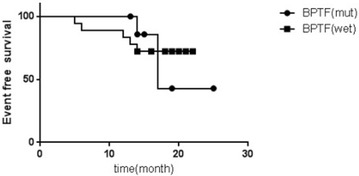
EFS between patients with BPFT mutation and wild type: after the 2nd and 4th chemotherapy, the tumor volume reduced and the BPTF gene mutation rate decreased accordingly. Patients with BPTF mutation may have a worse prognosis compared with patients with wild type, but the difference is not significant (P = 0.352)

Based on our WES data, through the continuous monitoring in the process of the treatment of 27 patients, we identified recurrent alterations at trios included mutations in the bromodomain PHD finger transcription factor (BPTF) and transmembrane and coiled-coil domains 3 (TMCO3). Six recurrent somatic mutations in BPTF were identified in nine patients after filtering nails data, including three missense mutation and three noncoding region mutation. c.*231A>G (exon 28); c.*1592C>A (exon 28); c.*983C>A (exon 28); c.5691C>G (exon 14); c.6311C>A (exon 19); c.247G>C (exon 1). Correlation analysis showed that the BPTF gene mutation rate was in positive correlation with tumor volume during chemotherapy (CC = 0.428, P = 0.021) (Fig. 1). After the 2nd and 4 th chemotherapy, the tumor volume reduced and the BPTF gene mutation rate decreased accordingly. Patients with BPTF mutation may have a worse prognosis compared with patients with wild type, but the difference is not significant (P = 0.352) (Fig. 2).

## Novel somatic mutations indicated chemo-therapy resistant in NB patients

By WES sequencing “diagnosis-2nd chemotherapy-4th chemotherapy” trios, recurrent new mutations in cell growth regulator CGREF1, transcriptional factor CUX2, diphosphohydrolase ENTPD2, solute carrier family 45 SLC45A1, and the component of RNA spliceosome TRA2A were identified as the novel somatic mutation during the chemotherapy. SLC45A1 was isolated initially from a region on chromosome 1p that is frequently deleted in human neuroblastoma. CGREF1, CUX2 and ENTPD2 are involved in mediating neural cell growth, as well.

## Baseline research of plasma cfDNA and bone marrow gDNA in three NB patients

**Fig. 3:**
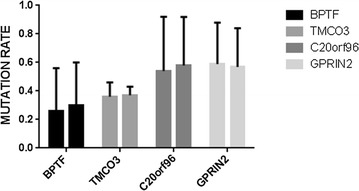
Correlations of BPTF, TMCO3, GPRIN2, and C20orf96 mutation rate of plasma cfDNA, and bone marrow gDNA: correlation analysis showed that the mutation rate of plasma cfDNA were in positive correlation with gDNA of bone marrow (P = 0.001)

The cfDNA is dissociated DNA that is circled in the bloodstream. It can be captured as a biological sample such as plasma or serum for disease analysis and is suitable for a range of research applications such as PCR, and next-generation sequencing. When we performed the baseline research, both cfDNA from plasma and gDNA from bone marrow blood were used for WES. We found that the mutations in BPTF, TMCO3, as well as G protein regulated inducer of neurite outgrowth 2, GPRIN2, and chromosome 20 open reading frame 96, C20orf96, were highly consistent identified in bone marrow blood white cells and plasma cfDNA. Correlation analysis show that the above four genes mutation rates in plasma cfDNA were in positive correlation with bone marrow gDNA (P = 0.001). Figure 3 shows the mutation rate of BPTF, TMCO3, GPRIN2 and C20orf96 in the plasma and bone marrow. Our results demonstrated that plasma cfDNA might be a new option for MRD monitoring and the sensitivity of next generation sequencing is much higher than that of traditional bone marrow smear.

## Discussion

NB originals from the neural crest precursor cells of the developing sympathetic nervous system and is remarkably heterogenous in its malignant potential with diverse clinical presentations, courses, and overall prognosis. NB with higher stage disease remains to be incurable with survival rates less than 50%. There is no common neuroblastoma-specific genomic alteration were uncovered [1, 2].

Screening gene variation in the process of therapy could provide us better and earlier information than the traditional imaging examination [6–8]. To determine tumor specific biomarkers for valuing the effect of chemotherapy in NB individuals, we focus on gene which its mutation rate changes consistent with tumor size variation.

Several novel somatic mutations were identified in all three trios comply or against the trendy of tumor size variation. Six recurrent mutations in BPTF were identified in NB patients under the continuous monitoring. BPTF gene codes nucleosome-remodeling factor subunit containing a DNA-binding domain and a zinc finger motif, indicating that BPTF might play a role in the regulation of transcription. High levels of BPTF were detected in fetal brain and in patients with neurodegenerative diseases. The gain of function mutation of BPTF could confer the abnormal expression itself and activate a set of target genes to contribute to the tumorigenesis and maintenance of NB [9–12]. Notably, BPFT gene mutation, were uncovered in the same trendy. Variation of the mutation rate in BPTF was coinciding with tumor size changes, indicating that there is strong positive relation between the mutations and prognosis. Our finding could uncover potential biomarkers of chemotherapy evaluation.

BPTF and TMCO3 mutations were detected at the time of diagnosis, after chemotherapy, BPTF mutation rate decreased, together with C20orf96 and LOC100996470, indicating tumor burden reduced, and BPTF may contribute to bone marrow metastasis.

Therapy-resistant relapse is the main cause of NB mortality. In this investigation, we identified several novel somatic mutations by WES sequencing “diagnosis-2nd chemotherapy-4th chemotherapy” trios. Recurrent new mutations in cell growth regulator CGREF1, transcriptional factor CUX2, diphosphohydrolase ENTPD2, solute carrier family 45 SLC45A1, and the component of RNA spliceosome TRA2A were identified as the novel somatic mutation during the chemotherapy. SLC45A1 was isolated initially from a region on chromosome 1p that is frequently deleted in human neuroblastoma. CGREF1, CUX2 and ENTPD2 are involved in mediating neural cell growth, as well [13–16]. The emergence of these mutations may indicate chemo-therapy resistant in NB patients.

When we look at the mutations detected in plasma cfDNA, we found that the mutations were highly consistent identified in bone marrow blood white cells and plasma cfDNA. Our results demonstrated that plasma cfDNA maybe a new option for MRD monitoring and the sensitivity of next generation sequencing is much higher than that of traditional bone marrow smear.

Consider that the limitation of sample size, we elevated the criteria of filtering, only the mutations shared by all patients remained maybe that is the reason we didn’t figure out the well-known mutations such as KRAS and NRAS. Only one recurrent mutation on MLL4 was identified in all the three samples when we do the baseline research, MLL4, lysine (K)-specific methyltransferase was the shared somatic mutation. The missense mutation was predicted to destroy protein structure by PROVEN software. MLL4 is a histone methyltransferase which methylates Lys-4 of histone H3 (H3K4me). H3K4me1/3 represents a specific tag for epigenetic transcriptional activation. Acts as a coactivator for estrogen receptor by being recruited by ESR1, thereby activating transcription. Abolish MLL4 function could impair the expression of a subset target genes. Sharing this mutation in the individuals with NB may indicate the dysregulation in the transcriptome in this malignancy tumor.

In the continuous screening genetic alterations, there are several mutation appear or disappear following the tumor size changes. Although due to the limitation of sample size, we missed some of molecular marker indicating the effects of therapy, the stricter filtering criteria allow us to identify the meaningful mutilations. This strategy could make sense to identify therapy specific genetic alteration in NB patients. Further, we ought to scale up the sample and preform functional validation of the mutations.

## Conclusion

WES allows for novel mutation detection at low cost, could therefore be routinely applied clinically and reveal reliable bio-markers, has the potential for accelerating the personalized detection, therapy and monitoring of tumor. We anticipate that a panel of markers, such as BPTF, TMCO3, CUX2, combined with classic markers: PHOX2B, TH, DCX, will prove valuable in a variety of clinical settings, including the assessment of tumor, drug resistance, residual disease monitoring and prediction of recurrence.

## Additional file

**Additional file 1.** Original data of 27 NB patients of clinical features and NGS results.

## Abbreviations

BPTF: bromodomain PHD finger transcription factor
cfDNA: cell free DNA
C20orf96: chromosome 20 open reading frame 96
CGREF1: cell growth regulator with EF-hand domain 1
ENTPD2: ectonucleoside triphosphate diphosphohydrolase 2
gDNA: genomic DNA
GPRIN2: G protein regulated inducer of neurite outgrowth 2
INSS: international neuroblastoma staging system
MRD: minimal residual disease
NB: neuroblastoma
NGS: next generation sequencing
SLC45A1: solute carrier family 45 member 1
TMCO3: transmembrane and coiled-coil domain 3
TRA2A: transformer-2 protein homolog A
WES: whole exome sequencing
